# Aprotinin May Increase Mortality in Low and Intermediate Risk but Not in High Risk Cardiac Surgical Patients Compared to Tranexamic Acid and ε-Aminocaproic Acid – A Meta-Analysis of Randomised and Observational Trials of over 30.000 Patients

**DOI:** 10.1371/journal.pone.0058009

**Published:** 2013-03-06

**Authors:** Patrick Meybohm, Eva Herrmann, Julia Nierhoff, Kai Zacharowski

**Affiliations:** 1 Clinic of Anesthesiology, Intensive Care Medicine and Pain Therapy, University Hospital Frankfurt, Frankfurt am Main, Germany; 2 Institute of Biostatistics and Mathematical Modelling, University Hospital Frankfurt, Frankfurt am Main, Germany; University of Colorado Denver, United States of America

## Abstract

**Background:**

To compare the effect of aprotinin with the effect of lysine analogues (tranexamic acid and ε-aminocaproic acid) on early mortality in three subgroups of patients: low, intermediate and high risk of cardiac surgery.

**Methods and Findings:**

We performed a meta-analysis of randomised controlled trials and observational with the following data sources: Medline, Cochrane Library, and reference lists of identified articles. The primary outcome measure was early (in-hospital/30-day) mortality. The secondary outcome measures were any transfusion of packed red blood cells within 24 hours after surgery, any re-operation for bleeding or massive bleeding, and acute renal dysfunction or failure within the selected cited publications, respectively.

Out of 328 search results, 31 studies (15 trials and 16 observational studies) included 33,501 patients. Early mortality was significantly increased after aprotinin vs. lysine analogues with a pooled risk ratio (95% CI) of 1.58 (1.13–2.21), p<0.001 in the low (n = 14,297) and in the intermediate risk subgroup (1.42 (1.09–1.84), p<0.001; n = 14,427), respectively. Contrarily, in the subgroup of high risk patients (n = 4,777), the risk for mortality did not differ significantly between aprotinin and lysine analogues (1.03 (0.67–1.58), p = 0.90).

**Conclusion:**

Aprotinin may be associated with an increased risk of mortality in low and intermediate risk cardiac surgery, but presumably may has no effect on early mortality in a subgroup of high risk cardiac surgery compared to lysine analogues. Thus, decisions to re-license aprotinin in lower risk patients should critically be debated. In contrast, aprotinin might probably be beneficial in high risk cardiac surgery as it reduces risk of transfusion and bleeding complications.

## Introduction

Excessive postoperative bleeding after cardiac surgery increases transfusion requirements, which is associated with postoperative infections and ischaemic events [1]. A recent systematic review from the Cochrane Collaboration have demonstrated that aprotinin is the most effective drug in decreasing perioperative bleeding and the need for blood transfusion and re-operation [2]. At the end of 2007, however, worldwide marketing of aprotinin was suspended as the findings from the Blood Conservation using Antifibrinolytics Trial (BART) suggested a trend towards increased 30-day mortality in the aprotinin treatment arm despite a modest reduction in the risk of massive bleeding [3]. As a consequence, use of tranexamic acid and aminocaproic acid increased as alternative antifibrinolytic agents worldwide, although concerns are also increasing with regard to potential adverse effects [4]. Moreover, it is unclear if use of lysine analogues is adequate in patients at highest risk (the originally intended patient population by Royston et al. [5]) in whom prophylactic treatment with aprotinin may be of greatest benefit. In this respect, the European Medicines Agency's Committee for Medicinal Products for Human Use (CHMP) revisited its previous recommendation on aprotinin in February 2012. The CHMP concluded that the benefits of aprotinin outweigh its risk in appropriately managed patients undergoing isolated heart bypass surgery (not combined with other heart surgery), and recommended that the suspension of aprotinin medicines in the EU should be lifted for this revised indication.’ [6]

In a retrospective single-center cohort study, Karkouti et al. previously showed that aprotinin tends to have a better risk-benefit profile than tranexamic acid in high-risk, but not in low- to moderate-risk patients [7]. As a meta-analysis of only randomised trials [2] might be too small to provide precise estimates of early mortality, we performed a more complete assessment of the epidemiologic evidence and reviewed studies published since 1990, which have examined the association between aprotinin, tranexamic acid and ε-aminocaproic acid regarding early mortality following cardiac surgery. The study results of the primary endpoint are reported separately for randomised trials, adjusted and unadjusted observational studies, respectively.

## Methods

### Study identification

**Figure 1:**
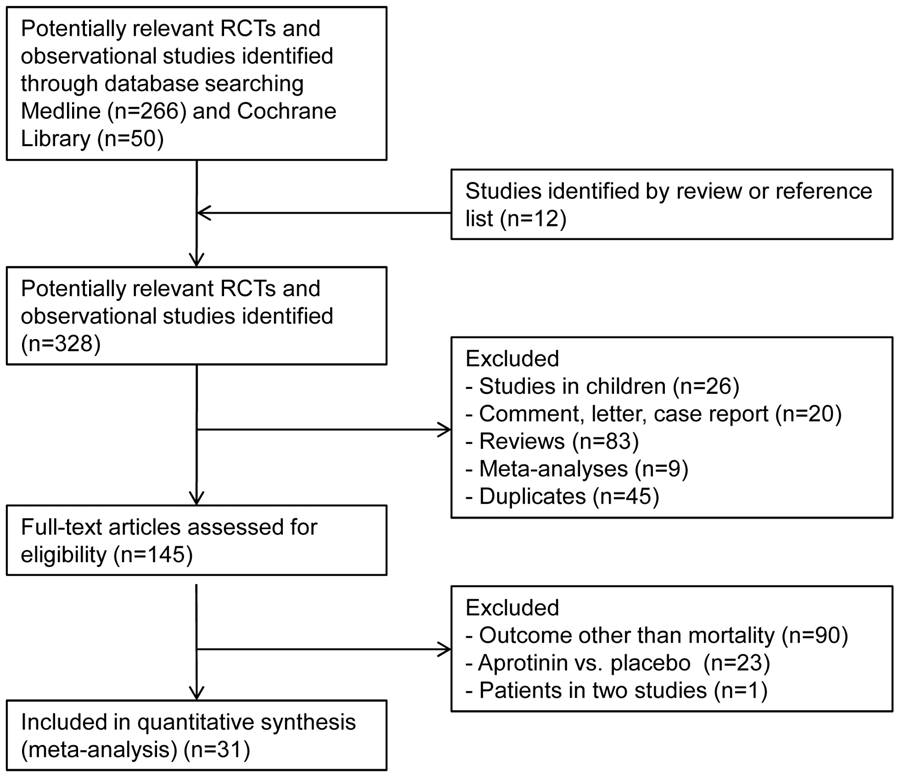
Flow of information through the different phases of the systemic review.

We undertook a systematic search of the literature to identify published reports which compared mortality after cardiac surgery for patients given aprotinin compared with tranexamic acid and ε-aminocaproic acid. We searched Medline using the following strategy: MeSH terms “Cardiac Surgical Procedures” and “Humans”, MeSH Major Topic “Aprotinin” with limits (a) publication date from 1^st^ January 1990 to 8^th^ April 2012, (b) studies in English, and (c) studies classified as a clinical trial, meta-analysis, randomised controlled trial, review, clinical trial, phase I, clinical trial, phase II, clinical trial, phase III, clinical trial, phase IV, comparative study, controlled clinical trial, corrected and republished article, evaluation studies, journal article, or multicenter study. A total of 266 Medline articles were identified and the abstracts were searched for reference to in-hospital mortality or mortality to 30-days after surgery. We also searched reference lists of identified articles and included 12 studies additionally [8], [9], [10], [11], [12], [13], [14], [15], [16], [17], [18], [19]. Additionally, we searched Cochrane Library using “Cardiac Surgical Procedures” and “Aprotinin” with publication date from 1^st^ January 1990 and found 50 articles in English. We excluded articles which only compared aprotinin to control (placebo), duplicates, studies which did not consider mortality and studies which reported long-term mortality, studies in children, meta-analyses, comments, case reports, and reviews (Fig. 1). Again, as a meta-analysis of only randomised trials (RCT) might be too small to provide precise estimates of early mortality, we performed a more complete assessment of the epidemiologic evidence, as it has recently been performed for other indications if randomized studies are insufficiently [20]. The study results of the primary endpoint are reported separately for RCT, adjusted and unadjusted observational studies. All original studies were abstracted by one reviewer unblinded to authors, institution and journal. We attempted to contact the authors of included studies and requested additional information in terms of mortality if this was not contained in published articles.

We compared aprotinin versus both lysine analogues (tranexamic acid and/or ε-aminocaproic acid) that were not separately analysed in our review, as the latest Cochrane review by Henry et al. [2] did not find any significant difference between both antifibrinolytics in terms of number of exposed allogeneic blood, re-operation for bleeding, mortality, and other adverse outcome events (myocardial infarction, stroke, deep vein thrombosis, or renal failure/dysfunction).

We defined a priori three subgroups of risk for bleeding:

1. Low risk surgery was predominantly defined as isolated coronary artery bypass graft (CABG) (or single valve surgery),
2. Intermediate risk surgery was predominantly defined as combined cardiac surgery, e.g. CABG with valve surgery,
3. High risk surgery was predominantly defined as complex surgery, e.g. redo sternotomy, multiple valve surgery, surgery of ascending aorta or aortic arch, or emergency surgery.

We also provide a systemic overview of all factors included in the regression analyses (Table S4 in File S1). Unfortunately, some studies did not allow allocating the events to a specific type of surgery. Therefore, the definition of low, intermediate or high risk surgery was based on the type of surgery that was mainly performed within the study, although heterogeneity of risk slightly varied between studies.

### Data extraction and quality assessment

All data with regard to authorship, year of publication, study design (RCT, observational study), study population (sample size, type of cardiac operation), length of follow-up and clinical endpoints were extracted. Methodological quality of the included studies was assessed using the Downs and Black Checklist for both RCT and observational trials [21]. The Downs and Black tool comprises six sections that assess reporting (total score: 11), external validity (total score: 3), internal validity bias (total score: 7), internal validity confounding (total score: 6), and power (total score: 2). A maximum score of 29 indicates the highest methodological quality and a score of zero represents the poorest methodological quality.

### *Endpoints*

The primary endpoint of the systematic review was overall early mortality. From all studies, we used 30-day mortality. If 30-day mortality was not reported, we used in-hospital mortality. Secondary endpoints were i) any transfusion of packed red blood cells within 24 hours after surgery, ii) any re-operation for bleeding or massive bleeding, and iii) acute renal dysfunction or failure within the 31 cited publications, respectively. We used definitions of acute renal dysfunction or acute renal failure as defined by the authors in their original papers. The presented studies are selected to report mortality data.

### Statistical analysis

The meta-analysis was done in line with recommendations from the Preferred Reporting Items for Systemic reviews and Meta-Analyses (PRISMA statement) [22] and with previous recommendations for reporting observational studies (MOOSE) [23]. In addition, we reported against the AMSTAR instrument in terms of the adequacy of conducting this review [24]. All analysis and graphical illustrations were conducted using R from the R Foundation for Statistical Computing, Vienna, Austria, particularly the R package meta by G. Schwarzer. Study protocol is provided in (File S2, File S3).

Risk ratio (RR) and 95% confidence intervals (95% CI) were calculated using the random effects model (DerSimonian and Laird estimator) [25]. Typically, studies with larger sample size received more weight when calculating the RR. RRs are undefined and excluded for studies with no event in either arm. For studies with zero events 0.5 is added to the corresponding cells. The presence of heterogeneity and comparisons of subgroups of trials was tested by Q-test and the results are given in the figures. To find a possible evidence for publication bias funnel plots of the RR were generated and asymmetry was tested by the rank correlation test based on Kendall's tau. We considered P<0.05 to be statistically significant.

## Results

A total of 31 published reports (15 trials and 16 observational studies) were identified. Detailed descriptions of these studies are given in (Table S1, S2, S3 in File S1). The majority of studies considered in-hospital mortality (n = 21) or mortality to 30-days (n = 9) whereas one study reported ‘postoperative mortality’ (n = 1) [13]. In terms of studies quality, the median Downs and Black score was 18.5 (range 12–27 points) considering the total number of 31 studies.

### Meta-analysis of primary endpoint

**Figure 2:**
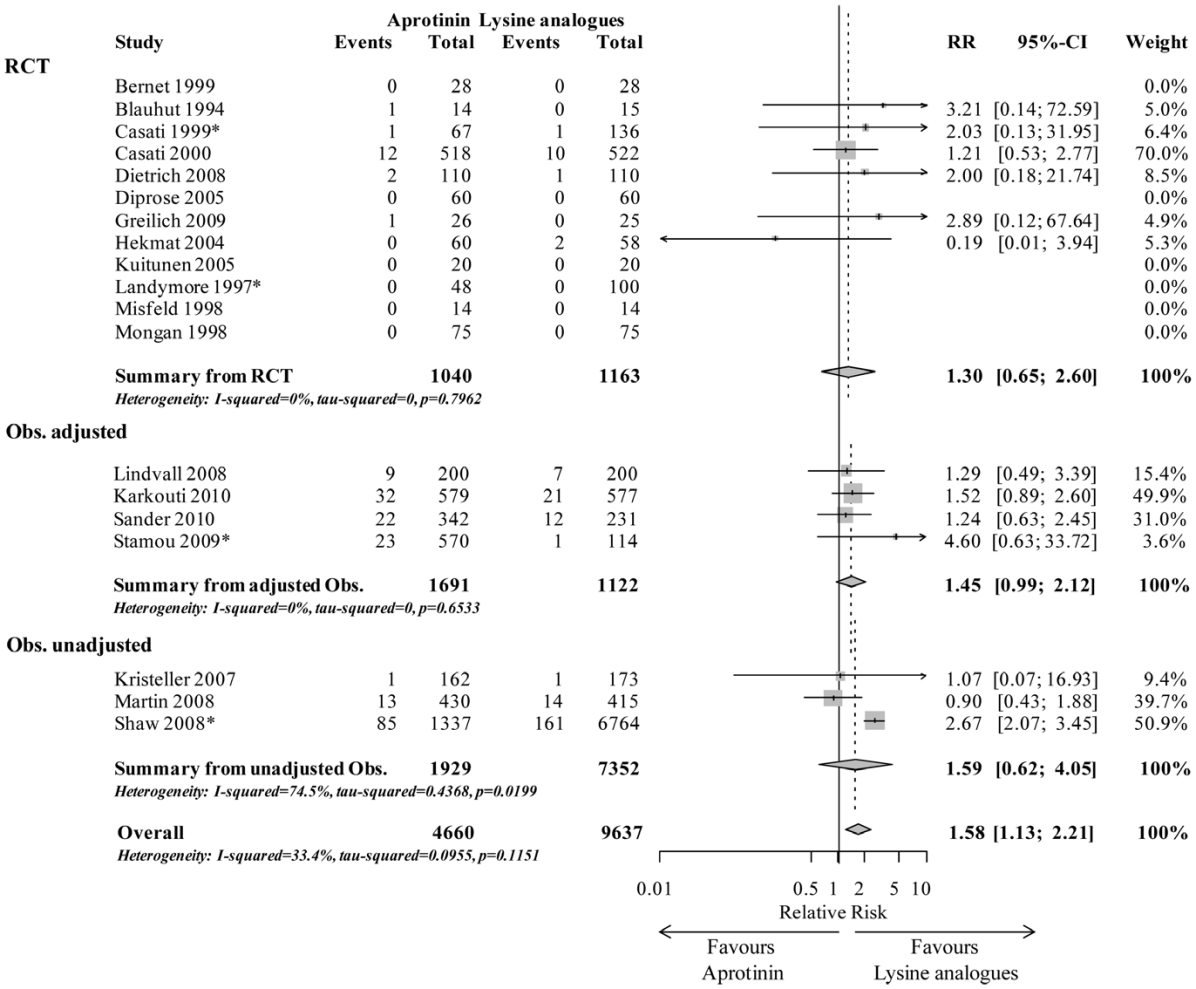
Early mortality in low risk surgery (subgroup 1). Forrest plot showing risk ratio (95% CI) of studies comparing aprotinin vs. lysine analogues (tranexamic acid and/or aminocaproic acid, indicated by *) for in-hospital/30-day mortality in a subgroup of low risk cardiac surgical patients sorted by randomised controlled trials (RCT), adjusted and unadjusted observational studies, respectively.

Analysing 14,297 patients with low risk, aprotinin was significantly associated with increased early mortality compared to lysine analogues (1.58 (1.13–2.21), p<0.001). The study results of the primary endpoint are displayed separately for RCT, adjusted and unadjusted observational studies in Fig. 2.

**Figure 3:**
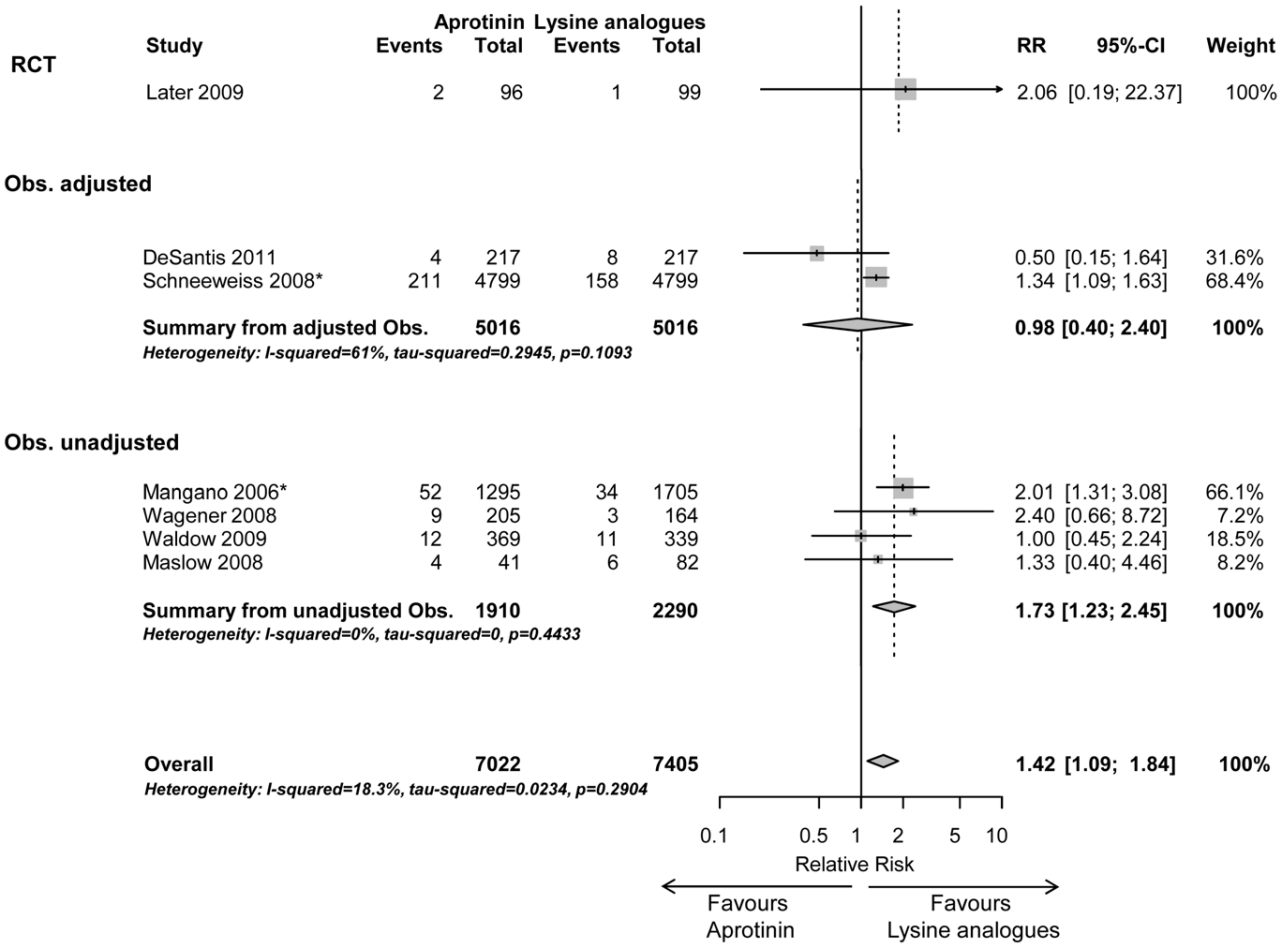
Early mortality in intermediate risk surgery (subgroup 2). Forrest plot showing risk ratio (95% CI) of studies comparing aprotinin vs. lysine analogues (tranexamic acid and/or aminocaproic acid, indicated by *) for in-hospital/30-day mortality in a subgroup of intermediate risk cardiac surgical patients sorted by randomised controlled trials (RCT), adjusted and unadjusted observational studies, respectively.

In patients with an intermediate risk (n = 14,427) risk ratio of mortality was significantly increased (1.42 (1.09–1.84), p<0.001) according to the Random effects model (Fig. 3).

**Figure 4:**
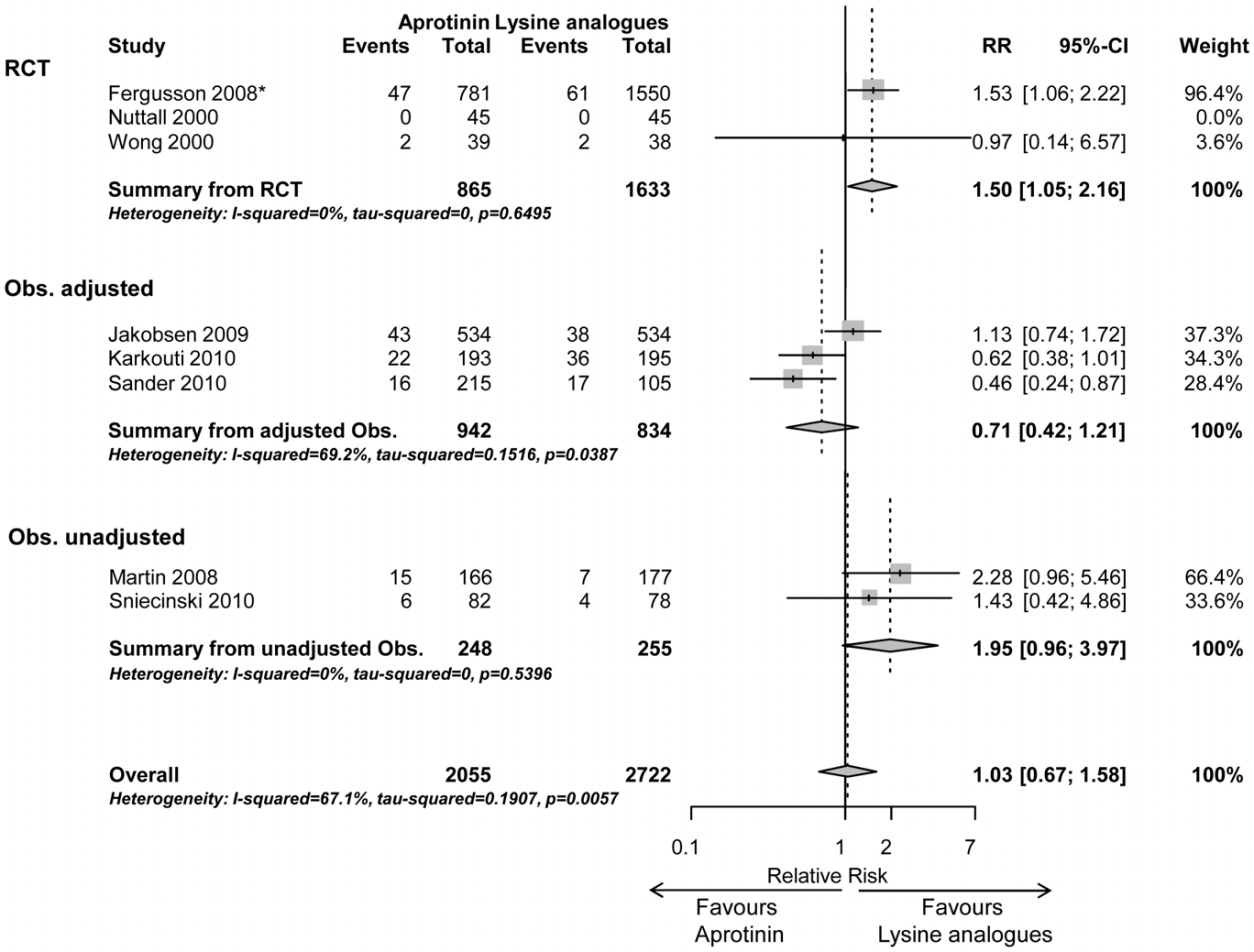
Early mortality in high risk surgery (subgroup 3). Forrest plot showing risk ratio (95% CI) of studies comparing aprotinin vs. lysine analogues (tranexamic acid and/or aminocaproic acid, indicated by *) for in-hospital/30-day mortality in a subgroup of high risk cardiac surgical patients sorted by randomised controlled trials (RCT), adjusted and unadjusted observational studies, respectively.

In the subgroup of high risk patients (n = 4,777), the risk for mortality did not differ significantly between aprotinin and lysine analogues (1.03 (0.67–1.58, p = 0.90) indicating a probably neutral effect for aprotinin compared to lysine analogues in terms of early mortality (Fig. 4).

Funnel plot analysis showed no obvious deviations from symmetry, so due to this results there are no indications of publication bias (Kendall's rank correlation with p>0.20 for all three risk groups; correlation coefficients: τ = 0.18 for the low risk group; τ = 0.24 for the intermediate risk group; τ = −0.24 for the high risk group).

Even so different results are obtained for low, intermediate and high risk patients, a direct comparison of RR between these three groups did not reach statistical significance (p = 0.11).

Note, that there are also no significant differences between trials focusing on in-hospital mortality or 30-days mortality (p = 0.23) justifying the combined analysis approach.

### *Meta-analysis of secondary endpoints (non-representative sample)*

Risk ratio for transfusion of red blood cells within 24 hours after surgery could be analysed in 18 studies and was 0.84 (0.74–0.96, p = 0.01, n = 3,565), 0.90 (0.76–1.05, p = 0.19, n = 752) and 0.79 (0.70–0.89, p<0.001, n = 3,809) in the low, intermediate and high risk subgroup, respectively (Fig. S1). Risk ratio for re-operation or any massive bleeding could be analysed in 21 studies and was 0.87 (0.66–1.14, p = 0.31, n = 5,515), 0.75 (0.09–6.03, p = 0.79, n = 752) and 0.79 (0.54–1.14, p = 0.20, n = 4,776), respectively (Fig. S2). Out of the 31 selected studies, 15 studies reported data on either acute renal dysfunction or acute renal failure showing that risk ratio did not significantly differ between aprotinin and lysine analogues in the low (1.35 (0.98–1.87), p = 0.07, n = 4,153), intermediate (1.10 (0.67–1.83), p = 0.70, n = 14,058) and high risk subgroup (1.19 (0.93–1.54), p = 0.17, n = 4,273), respectively (Fig. S3).

## Discussion

We address a highly topical issue [26], [27] – whether aprotinin is safe to use in certain patient groups?

To best of our knowledge, this is the first review stratifying cardiac surgical patients to their individual risk for bleeding and surgical complications, and demonstrating that aprotinin has diverse effects depending on the risk profile. Specifically, we found that aprotinin leads to higher mortality in low and intermediate risk, but presumably may not affect mortality in high risk cardiac surgical patients.

The suspension of aprotinin a few years ago has forced clinicians to find alternative blood-sparing agents for use during cardiac surgery. The two alternatives are the lysine analogues ε-aminocaproic acid which has no approval in Europe or Canada for human administration, and tranexamic acid which is now exclusively used in these countries. Nevertheless, a few number of problems with lysine analogues, in particular with tranexamic acid has emerged, since there is little evidence for a benefit of tranexamic acid to reduce transfusion burden, particularly in patients at higher risk for bleeding and transfusion.

Although our meta-analysis mainly focuses on early mortality as the primary endpoint, we also examined the effects of aprotinin vs. lysine analogues regarding acute renal dysfunction or failure as a non-representative sample. This endpoint was reported in 15 studies showing that risk ratio tended to be increased, but did not significantly differ between aprotinin and lysine analogues in the low, intermediate and high risk subgroup. Similarly, we recently found in a retrospective observational study including 9,875 cardiac surgical patients with propensity-adjusted, multivariate logistic regression [28], that aprotinin did not significantly increased risk of postoperative renal dysfunction in on-pump cardiac surgery. Further, the recent Cochrane review [2] including the ‘head-to-head’ BART study did not find any difference with any of the antifibrinolytic drugs in terms of kidney failure, myocardial infarction or stroke.

More interestingly, the recent Cochrane analysis also suggested beneficial effects of aprotinin by reducing risk of transfusion and bleeding complications. Moreover, the risk for perioperative use of blood products such as fresh frozen plasma and cryoprecipitate may have even increased in the post-aprotinin era [29]. In the selected studies of our meta-analysis, aprotinin was associated with a reduced risk for transfusion of red blood cells, and tended to reduce risk for re-operation or any massive bleeding irrespective of the surgical risk.

In this respect, the European Medicines Agency recommended lifting suspension of aprotinin at the 17^th^ February 2012 as benefits (less transfusion requirements, less bleeding-associated harm) outweigh risks (mortality) in restricted range of indications. Very surprisingly, ‘suspension was lifted in appropriately managed patients with isolated heart bypass surgery.’ Our present meta-analysis, however, does not support this recommendation, as aprotinin was associated with a significant increased risk of early mortality in both subgroups - low risk and intermediate risk patients. Contrarily, aprotinin presumably may not affect mortality in higher risk surgical patients undergoing complex surgery who have a higher risk of life threatening haemorrhage and, consequently, of needing blood transfusion. Aprotinin's ability to decrease the risk of transfusion of red blood cells more than tranexamic acid and aminocaproic acid has repetitively been proven in most of the studies [2], [14], [29], [30]. The clinical implication of our findings is that aprotinin may be the antifibrinolytic of choice and should therefore remain available for clinical use in these high risk cardiac surgical patients. These are the patients with multiple co-morbidities who are undergoing emergency, redo sternotomy, or complex procedures that require prolonged cardio-pulmonary bypass support, e.g. multiple valve surgery, or surgery of ascending aorta or aortic arch with hypothermic cardiac arrest. The propensity matched paired analysis by Karkouti et al. [7] identified patients whose risk status placed them to the top 10th percentile of their institution's cardiac surgery population. These implications are also supported by a subgroup analysis of the BART which revealed that aprotinin did not affect early mortality in elderly patients and patients with high co-morbidity as relative risk decreased with older age (age<65 years: 3.42 (1.14–10.26) vs. age>80 years: 0.67 (0.26–1.74)), co-existing morbidity (none: 4.40 (1.28–15.15) vs. co-morbid: 1.24 (0.76–2.03), and higher American Society of Anesthesiologist (ASA) physical status class (ASA class <4: 2.18 (0.95–5.04) vs. ASA class ≥4 points: 1.34 (0.78–2.32)).

This review has also some limitations. First, the definition of low, intermediate or high risk surgery was based on the type of surgery that was mainly performed within the respective study, although the heterogeneity in risk slightly varied between studies. Unfortunately, some studies did not allow allocating the events to a specific type of surgery. Secondly, as our present meta-analysis mainly focussed on early mortality as the primary endpoint, analysis of secondary endpoints underlay a study selection bias. Based on the 31 selected studies, the results are descriptive and non-representative. Data from the latest Cochrane review including only RCTs [2], however, suggested a significant benefit of aprotinin over the lysine analogues tranexamic acid and ε-aminocaproic acid in terms of i) reducing perioperative blood loss, ii) reducing the need for RBC transfusion, and iii) reducing the need for re-operation due to bleeding, respectively. In addition, a problem is lack of large prospective randomised studies. We included 15 RCTs, but even the two largest trials by Casati et al. [31] and Fergusson et al. [3] that each included more than 1,000 patients did not focus on mortality as the primary endpoint, respectively. The application of formal meta-analytic methods to observational studies and cross design synthesis has been controversial. One reason for this has been that potential biases in the original studies make the calculation of a single summary estimate of effect of exposure potentially misleading [23]. Large observational studies, which have residual confounding, can swamp smaller well controlled randomised trials during data pooling. Nevertheless, we performed a complete assessment of the epidemiologic evidence, as a meta-analysis of only randomised trials would be too small to provide precise estimates of early mortality. Taking into account these methodological limitations, we reported mortality results separately for RCT, adjusted and unadjusted observational studies, respectively.

Moreover, the search was limited to published reports in Medline and Cochrane Library, and the authors did not ask experts for additional unpublished reports.

## Conclusions

First, aprotinin may be associated with increased risk of early mortality in low and intermediate risk cardiac surgical patients, in particular in patients with mainly isolated CABG or CABG combined with valve surgery. Therefore, the recent recommendation of the European Medicines Agency lifting suspension of aprotinin in lower risk patients should critically be debated. Secondly, based on the known beneficial effects of aprotinin reducing risk of transfusion and bleeding complications as well as the presumably neutral effect on mortality in high risk surgery, our findings suggest that aprotinin may be warranted in high risk patients, as determined by their co-morbidities, surgical acuity, and complexity. Given the observed incidence of mortality and the strong selection of high risk patients, however, an extremely large sample size would be required for a prospective randomised trial.

## Supporting Information

File S1 **Table S1, S2, S3, S4.** Details of published studies with low/intermediate/high risk surgery of in-hospital/30-day mortality after cardiac surgery (1990–2012).
(DOCX)

File S2 **Study protocol for a prospective meta-analysis.**
(DOCX)

File S3 **Study protocol appendix.**
(DOCX)

Figure S1 **Risk ratio for transfusion of red blood cells within 24 hours after surgery.** Forrest plot showing risk ratio (95% CI) of studies comparing aprotinin vs. lysine analogues (tranexamic acid and/or aminocaproic acid, indicated by *) for transfusion of red blood cells within 24 hours after surgery in a subgroup of low (a), intermediate (b) and high risk (c) cardiac surgical patients, respectively.
(PDF)

Figure S2 **Risk ratio for re-operation or any massive bleeding.** Forrest plot showing risk ratio (95% CI) of studies comparing aprotinin vs. lysine analogues (tranexamic acid and/or aminocaproic acid, indicated by *) for re-operation or any massive bleeding in a subgroup of low (a), intermediate (b) and high risk (c) cardiac surgical patients, respectively.
(PDF)

Figure S3 **Risk ratio for acute renal dysfunction or acute renal failure.** Forrest plot showing risk ratio (95% CI) of studies comparing aprotinin vs. lysine analogues (tranexamic acid and/or aminocaproic acid, indicated by *) for acute renal dysfunction or acute renal failure in a subgroup of low (a), intermediate (b) and high risk (c) cardiac surgical patients, respectively. Please note that definition of acute renal dysfunction and acute renal failure varied moderately between studies.
(PDF)
