# Supplementary material for: Aprotinin May Increase Mortality in Low and Intermediate Risk but Not in High Risk Cardiac Surgical Patients Compared to Tranexamic Acid and ε-Aminocaproic Acid – A Meta-Analysis of Randomised and Observational Trials of over 30.000 Patients
Source: PLoS One. 2013 Mar 6;8(3):e58009. doi: 10.1371/journal.pone.0058009 (PMC3590293; doi:10.1371/journal.pone.0058009)
Supplement: File S2 — Study protocol for a prospective meta-analysis. (DOCX) [file pone.0058009.s002.docx]

# Text S1-Study protocol for a prospective meta-analysis

# Effects of aprotinin on in-hospital and 30-day mortality in cardiac surgical patients

# compared to lysine analogues

# Objectives, eligibility and outcomes

A recent systematic review from the Cochrane Collaboration have demonstrated that aprotinin is the most effective drug in decreasing perioperative bleeding and the need for blood transfusion and re-operation.[1] At the end of 2007, however, worldwide marketing of aprotinin was suspended as the findings from the Blood Conservation using Antifibrinolytics Trial (BART) suggested a trend towards increased 30-day mortality in the aprotinin treatment arm despite a modest reduction in the risk of massive bleeding.[2] Nevertheless, use of alternative antifibrinolytic agents (e.g. tranexamic acid and aminocaproic acid) is also associated with potential adverse effects.[3] Most importantly, it is unclear if use of lysine analogues is adequate in patients at highest risk (the originally intended patient population by Royston et al.[4]) in whom prophylactic treatment with aprotinin may be of greatest benefit.

In a retrospective single-center cohort study, Karkouti et al. previously showed that aprotinin tends to have a better risk-benefit profile than tranexamic acid in high-risk, but not in low- to moderate-risk patients.[5] As a meta-analysis of only randomised trials[1] might be too small to provide precise estimates of early mortality, we aim at a more complete assessment of the epidemiologic evidence and will review studies, which have examined the association between aprotinin and lysine analogues regarding early mortality following cardiac surgery.

We hypothesise that aprotinin may have diverse effects in high risk compared to lower risk cardiac surgical patients in terms of early mortality.

# The present meta-analysis will focus on early mortality as the primary endpoint in cardiac surgical patients. As the current number of large prospective randomized studies is limited, and even the two largest trials by Casati et al.[6] and Fergusson et al.[2] that each included more than 1,000 patients did not focus on mortality as the primary endpoint, the planned meta-analysis will include both randomized and observational studies. The application of formal meta-analytic methods to observational studies has been controversial, as potential biases in the original studies make the calculation of a single summary estimate of effect of exposure potentially misleading.[7] Nevertheless, as a meta-analysis of only randomised trials would be too small to provide precise estimates of early mortality, a complete assessment of the epidemiologic evidence will be performed comparing aprotinin vs. tranexamic acid in a first step.

# *Revision (1^st^ May 2012):* As aminocaproic acid is very similar to tranexamic acid, and in some studies the same patient population was used, studies comparing aprotinin vs. aminocaproic acid will be added in the revised analysis. Also, data from both lysine analogues will be pooled, as the latest Cochrane review by Henry et al. 2011[1] did not find any significant difference between both antifibrinolytics in terms of number of exposed allogeneic blood, re-operation for bleeding, mortality, and other adverse outcome events (myocardial infarction, stroke, deep vein thrombosis, renal failure/dysfunction), respectively.

# As many observational studies show variable adjustment for confounding factors or propensity score analysis, we therefore will include each observational study irrespective of adjustment. Then, we will assign studies into 3 categories: 1) randomised controlled trials (RCT), 2) observational studies which attempted to match or adjust for confounding variables, and 3) observational studies which did not attempt to match or adjust for confounding variables. Mortality results will be reported separately for RCTs, adjusted and unadjusted observational studies, respectively. To our mind, a mixture of RCTs and retrospective non-randomized might be feasible to gain a more complete assessment of the evidence evaluating the risks of aprotinin to understand the drug’s safety profile.

Outcomes

The primary endpoint is overall early mortality. Generally, 30-day mortality will be used. If 30-day mortality is not reported, in-hospital mortality will be used.

Secondary endpoints will be i) any transfusion of packed red blood cells within 24 hours after surgery, ii) any re-operation for bleeding or massive bleeding, and iii) acute renal dysfunction or failure within the selected publications, respectively.

# Details of subgroups

In a retrospective single-center cohort study, Karkouti et al. showed that aprotinin tends to have a better risk-benefit profile than tranexamic acid in high-risk, but not in low- to moderate-risk patients [5]. Therefore, study patients will be divided into low-to-moderate and high risk cardiac surgery (high co-morbidity and complex surgery) in a first step.

*Revision (1^st^ May 2012):* Within the first version, heterogeneity in risk seemed wide within the low-to-moderate risk group, therefore, definition of risk profile will be revised and patients will subsequently be divided into 3 subgroups of risk:

1) Low risk surgery - predominantly defined as isolated CABG (or single valve surgery),

2) Intermediate risk surgery - predominantly defined as combined cardiac surgery, e.g. CABG with valve surgery,

3) High risk surgery - predominantly defined as complex surgery, e.g. redo sternotomy, multiple valve surgery, surgery of ascending aorta or aortic arch, or emergency surgery.

# Search methods

PubMed search will be used with the following strategy: MeSH terms "Cardiac Surgical Procedures” and “Humans”, MeSH Major Topic "Aprotinin" with limits (a) publication date from I January 2006 to 31 January 2012, (b) studies in English, and (c) studies classified as a clinical trial, meta-analysis, randomized controlled trial, review, clinical trial, phase I, clinical trial, phase II, clinical trial, phase III, clinical trial, phase IV, comparative study, controlled clinical trial, corrected and republished article, evaluation studies, journal article, or multicenter study.

# Unpublished ongoing trials with currently recruiting patients will not be considered.

The following exclusion criteria will be considered in a first step: 1) studies which only compared aprotinin either to control (placebo), 2) studies which did not consider mortality, 3) studies which reported long-term mortality, 4) studies in children, and 5) observational studies which did not attempt to match or adjust for confounding variables.

All original studies will be abstracted by one reviewer. We attempt to contact the authors of included studies and to request additional information not contained in published articles.

# *Revision (1^st^ May 2012):* Because there was a reasonably low event rate in the first version of the meta-analysis, criteria for study selection will be extended: i) publication date will be extended from 1^st^ January 1990 to 8^th^ April 2012, ii) search will be extended to comprehensive review articles and reference lists of identified articles (revealing 12 more studies), and iii) the initial exclusion criteria (excluding observational studies which did not attempt to match or adjust for confounding variables) will be deleted.

*Revision (17^th^ July 2012):* To adjust for studies not listed in Medline, literature search will further be extended to Cochrane Library using "Cardiac Surgical Procedures” and "Aprotinin" with publication date from 1^st^ January 1990 that revealed 50 more articles in English.

# Data collection and analysis

The planned meta-analysis will be done in line with recommendations from the Preferred Reporting Items for Systemic reviews and Meta-Analyses (PRISMA statement)[8] and with previous recommendations for reporting observational studies (MOOSE).[7] All analysis and graphical illustrations will be conducted using R from the R Foundation for Statistical Computing, Vienna, Austria, particularly the R package meta by G. Schwarzer. Risk ratio (RR) and 95% confidence intervals (95% CI) will be calculated using the random effects model (DerSimonian and Laird estimator).[9] Typically, studies with larger sample size will receive more weight when calculating the RR. RRs will be undefined and excluded for studies with no event in either arm. The presence of heterogeneity and comparisons of subgroups of trials will be tested by Q-test. The presence of publication bias will be assessed by generating funnel plots of the RR and testing asymmetry by the rank correlation test based on Kendall’s tau. We will consider P< 0.05 to be statistically significant.

# It is intended to update the meta-analysis data at regular intervals via ongoing cycles of data collection (3 yearly).

# Trial details

# Details of initially included studies are displayed in Appendix Table A1.

# Findings: Out of 114 search results, 9 studies (1 trial, 1 meta-analysis, and 7 observational studies) were considered. In the low-to-moderate risk subgroup (n=6,431 patients), early mortality was significantly increased in the aprotonin group with a pooled risk ratio (95% CI) of 1.36 (1.04-1.77, p<0.05). Contrarily, in the subgroup of high risk patients (n=3,967) the pooled estimated risk for mortality was 0.88 (0.61-1.25).

*Revision (1^st^ May 2012):* The fundamental problem of the first version is lack of data with a low event rate regarding mortality (244 events in the aprotinin group, 217 events in the tranexamic acid group).

Revised search strategy revealed a total of 266 articles (Fig. A1). Details of included studies are displayed in Appendix Table A2-A4.

Figure 1A. Flow of information through the different phases of the systemic review (revised 8^th^ May).

Findings: Out of 278 search results, 31 studies (15 trials and 16 observational studies) included 33,501 patients. Compared to the first version, 647 events were now identified in the aprotinin group and 617 events in the lysine analogues group. Early mortality was significantly increased after aprotinin vs. lysine analogues with a pooled risk ratio (95% CI) of 1.58 (1.13 – 2.21), p<0.001 in the low (n= 14,297) and of 1.42 (1.09 – 1.84), p<0.001 in the intermediate risk subgroup (n= 14,427), respectively. Contrarily, in the subgroup of high risk patients (n=4,777), the pooled estimated risk for mortality did not differ significantly between aprotinin and lysine analogues (1.03 (0.67 – 1.58), p=0.896).

*Revision (7^th^ July 2012):* Adding Cochrane search results in a revised version, 328 studies were identified (Fig. A2)

Figure 2A. Flow of information through the different phases of the systemic review (7^th^ July)

Findings: Out of 328 search results, again 31 studies (15 trials and 16 observational studies) included 33,501 patients. Early mortality was significantly increased after aprotinin vs. lysine analogues with a pooled risk ratio (95% CI) of 1.58 (1.13 – 2.21), p<0.001 in the low (n= 14,297) and in the intermediate risk subgroup (1.42 (1.09 – 1.84), p<0.001; n= 14,427), respectively. Contrarily, in the subgroup of high risk patients (n=4,777), the risk for mortality did not differ significantly between aprotinin and lysine analogues (1.03 (0.67 – 1.58), p=0.90).

Funnel plot analysis showed no obvious deviations from symmetry so due to this results there are no indications of publication bias (Kendall’s rank correlation with p>0.20 for all three risk groups; correlation coefficients: τ=0.18 for the low risk group; τ=0.24 for the intermediate risk group; τ=-0.24 for the high risk group).

Even so different results are obtained for low, intermediate and high risk patients, a direct comparison of RR between these three groups did not reach statistical significance (p=0.11).

Note, that there are also no significant differences between trials focusing on in-hospital mortality or 30-days mortality (p=0.23) justifying the combined analysis approach.

# Management and Co-ordination

PD Dr. Patrick Meybohm (PM), M.D., Prof. Dr. Dr. Kai Zacharowski (KZ), M.D., Ph.D., FRCA;

Clinic of Anesthesiology, Intensive Care Medicine and Pain Therapy, University Hospital Frankfurt, Theodor-Stern-Kai 7, 60590 Frankfurt am Main, Germany

Statistics: Prof. Dr. Eva Herrmann (EH), Ph.D., Julia Nierhoff (JN), M.Sc.;

Institute of Biostatistics and Mathematical Modelling, University Hospital Frankfurt, Theodor-Stern-Kai 7, 60590 Frankfurt am Main, Germany

Abstract will be reviewed by PM. Conception and design of the study, analysis and interpretation of data, drafting the article and final approval of the version to be submitted will be done by PM, KZ, EH, and JN. EH and JN will take responsibility for the integrity of the data and the accuracy of the data and statistical analyses.

**References**

1. Henry DA, Carless PA, Moxey AJ, O'Connell D, Stokes BJ, et al. (2011) Anti-fibrinolytic use for minimising perioperative allogeneic blood transfusion. Cochrane Database Syst Rev: CD001886.

2. Fergusson DA, Hebert PC, Mazer CD, Fremes S, MacAdams C, et al. (2008) A comparison of aprotinin and lysine analogues in high-risk cardiac surgery. N Engl J Med 358: 2319-2331.

3. Murkin JM, Falter F, Granton J, Young B, Burt C, et al. (2010) High-dose tranexamic Acid is associated with nonischemic clinical seizures in cardiac surgical patients. Anesth Analg 110: 350-353.

4. Royston D, Bidstrup BP, Taylor KM, Sapsford RN (1987) Effect of aprotinin on need for blood transfusion after repeat open-heart surgery. Lancet 2: 1289-1291.

5. Karkouti K, Wijeysundera DN, Yau TM, McCluskey SA, Tait G, et al. (2010) The risk-benefit profile of aprotinin versus tranexamic acid in cardiac surgery. Anesth Analg 110: 21-29.

6. Casati V, Guzzon D, Oppizzi M, Bellotti F, Franco A, et al. (2000) Tranexamic acid compared with high-dose aprotinin in primary elective heart operations: effects on perioperative bleeding and allogeneic transfusions. J Thorac Cardiovasc Surg 120: 520-527.

7. Stroup DF, Berlin JA, Morton SC, Olkin I, Williamson GD, et al. (2000) Meta-analysis of observational studies in epidemiology: a proposal for reporting. Meta-analysis Of Observational Studies in Epidemiology (MOOSE) group. Jama 283: 2008-2012.

8. Moher D, Liberati A, Tetzlaff J, Altman DG (2009) Preferred reporting items for systematic reviews and meta-analyses: the PRISMA statement. Ann Intern Med 151: 264-269, W264.

9. DerSimonian R, Laird N (1986) Meta-analysis in clinical trials. Control Clin Trials 7: 177-188.
