# Supplementary material for: Aprotinin May Increase Mortality in Low and Intermediate Risk but Not in High Risk Cardiac Surgical Patients Compared to Tranexamic Acid and ε-Aminocaproic Acid – A Meta-Analysis of Randomised and Observational Trials of over 30.000 Patients
Source: PLoS One. 2013 Mar 6;8(3):e58009. doi: 10.1371/journal.pone.0058009 (PMC3590293; doi:10.1371/journal.pone.0058009)
Supplement: File S3 — Study protocol appendix. (DOCX) [file pone.0058009.s003.docx]

**Text S2_Study protocol appendix**

**Table A1: Details of published studies of mortality after cardiac surgery (2006-2012).**

| **Study** | **Population** | **Study design** | **Sample size** | **Inclusion criteria** | **Exclusion criteria** | **Interventions** | **Analysis method** | **Notes** |
| --- | --- | --- | --- | --- | --- | --- | --- | --- |
| Fergusson 2008[1] | Patients who underwent cardiac surgery with CPB with a wide range of risk  Aug 2002 –  Oct 2007 | RCT in 19 Canadian centres | 2,331 cases  n=781 aprotinin  n=770 tranexamic acid  n=780 aminocaproic acid | Redo surgery  Isolated mitral value surgery  CABG+valve  Multiple valve replacement or repair  Surgery of the ascending aorta or aortic arch | Isolated CABG  Isolated mitral valve repair or aortic valve replacement  Congenital heart surgery  Heart transplant  VAD implantation | Aprotinin  Tranexamic acid  Aminocaproic acid | Relative risk  Logistic regression | Two subgroups: low-to-moderate and high risk |
| Henry 2011[2] | Patients who underwent cardiac surgery  1950 –  July 2010 | Meta-analysis of 114 randomised controlled trials | 9,497cases in  84 aprotinin vs. placebo trials  3,983 cases in 18 aprotinin vs. tranexamic acid  Trials (15 trials with mortality data)  2,220 cases in 12 aprotinin vs. aminocaproic acid trials (5 trials with mortality data) |  |  | Control  Aprotinin  Tranexamic acid  Aminocaproic acid | Random effects analysis of relative risk | Results reported exclude non-cardiac surgery trials |
| Karkouti 2006[3] | Patients who underwent cardiac surgery with CPB  Jun 1999 –  Jun 2004 | Retrospective analysis of consecutive series from a single centre in Toronto, Canada | 10,949 considered  10,870 eligible  449 propensity matched pairs | Age>18 years  CABG with CPB  +/- other procedures | Did not receive no antifibrinolytic agent  Participation in antifibrinolytic drug study | Aprotinin  Tranexamic acid | Conditional logistic regression  Propensity matched pairs | Effect estimate not reported, but was derived from the raw data presented |
| Lindvall 2008[4] | Patients who underwent CABG for acute coronary syndrome  2001 - 2003 | Retrospective analysis of matched cohort from two centres in Sweden (one centre used aprotinin and the other tranexamic acid) | 2,018 cases  200 matched pairs analysed | First time isolated CABG for acute coronary syndrome | Clopidogrel stopped more than 5 days before surgery in centre using aprotinin | Aprotinin  Tranexamic acid | Fisher’s exact test | Effect estimate not reported, but was derived from the raw data presented |
| Jakobsen 2009[5] | Patients who underwent cardiac surgery with CPB  Jan 2003 –  Dec 2006 | Retrospective analysis of consecutive series from a single centre in Denmark | 3,586 cases  3,535 analysed  1,068 for propensity matched pairs | All types of cardiac surgery with CPB | Multiple procedures during study period  Patients with an invalid personal identifier | Aprotinin  Tranexamic acid | Multivariable logistic regression and propensity score adjustment | 30-day mortality were obtained from the Danish Civil Registration System |
| Karkouti 2010[6] | Patients who underwent cardiac surgery with CPB  Jan 2005 –  May 2008 | Retrospective analysis of consecutive series from a single centre in Canada | 15,534 cases  15,365 analysed  1,544 for propensity matched pairs | All types of cardiac surgery with CPB | Patients who did not receive aprotinin or tranexamic acid and those who participated in the BART trial | Aprotinin  Tranexamic acid | Multivariable logistic regression and propensity score adjustment | Results reported separately for two distinct subgroups (low/moderate and high risk) |
| Sander 2010[7] | Patients who underwent cardiac surgery with CPB  Jan 2006 –  end of 2006 | Retrospective analysis of consecutive series from a single centre in Germany | 900 cases  analysed:  557 aprotinin (Jan 2006- Jun 2006)  336 tranexamic acid  (from Jul 2006) | All types of cardiac surgery with CPB |  | Aprotinin  Tranexamic acid | Multivariable logistic regression  Subgroup analysis of high risk patients (open cardiac surgery) | Effect estimate was not reported, but was derived from the raw data presented |
| DeSantis 2011[8] | Patients who underwent cardiac surgery  Oct 2005 –  Oct 2008 | Retrospective analysis of consecutive series from a single centre in US | 781 cases  analysed:  325 aprotinin  206 tranexamic acid  250 aminocaproic acid | CABG with CPB +/- aortic or mitral valve replacement/repair  Valve surgery  Heart transplantation | Congenital surgery  LVAD implantation | Aprotinin  Lysine Analogues  (Tranexamic acid  or Aminocaproic acid) | Multivariable logistic regression and propensity score adjustment | Adjusted estimates relative risks |

***Revision (8^th^ May 2012)***

**Table A2: Details of published studies with low risk surgery of in-hospital/ 30-day mortality after cardiac surgery (1990-2012).**

| **Study** | **Study design** | **Sample size/ Intervention** | **Inclusion criteria/**  **Type of surgery** | **Exclusion criteria**  **(cardiac related)** | **Analysis method** | **Mortality** | **Risk** |
| --- | --- | --- | --- | --- | --- | --- | --- |
| Bernet 1999[9] | RCT single-center, double-blind, Switzerland | 70 cases  N= 28 aprotinin  N=28 tranexamic acid | Isolated CABG | Unstable angina | Chi-square  or Fisher exact test | No specified | Low |
| Blauhut 1994[10] | RCT single-center, Austria | 45 cases  N=14 aprotinin  N=14 tranexamic acid  N=14 control | Isolated CABG | Redo operation,  therapy with antiplatelets, heparin or oral anticoagulants, emergency surgery | Not specified | In-hospital | Low |
| Casati 1999[11] | RCT single-center, unblinded, Italy | 210 cases  N=67 aprotinin  N=70 tranexamic acid  N=66 ε-aminocaproic acid | Isolated CABG | EF< 35%, need of ventricular assist device for weaning from CPB | Chi-square  or Fisher exact test | In-hospital | Low |
| Casati 2000[12] | RCT single-center, unblended, Italy  Jun 1996-Jul 1997 | 1,040 cases  N=518 aprotinin  N=522 tranexamic acid | Isolated CABG (~75%),  single valve surgery (~25%) | Not specified | Chi-square  or Fisher exact test | In-hospital | Low |
| Dietrich 2008[13] | RCT single-center, double-blinded, Germany | 220 cases  N=110 aprotinin  N=110 tranexamic acid | Isolated CABG (~60%),  single aortic valve surgery (~40%) | Redo operation,  previous exposure to aprotinin, emergency operation | Chi-square  or Fisher exact test | In-hospital | Low |
| Diprose 2005[14] | RCT single-center, double-blinded, United Kingdom | 186 cases  N=60 aprotinin  N=60 tranexamic acid  N=60 control | Isolated CABG (~75%),  single valve surgery (~25%) | Redo operation.  combined surgery,  two or more antiplatelet therapies, emergency operation | Chi-square  or Fisher exact test | In-hospital | Low |
| Greilich 2009[15] | RCT single-center, double-blinded, US  Sep 1998-Jan 2001 | 81 cases  N=26 aprotinin  N=25 ε-aminocaproic acid  N=27 control | Isolated CABG | Emergency operation, LVEF<30% | Chi-square  or Fisher exact test | 30-day | Low |
| Hekmat 2004[16] | RCT single-center, double-blinded, Germany | 120 cases  N=60 aprotinin  N=58 tranexamic acid | Isolated CABG | Redo operation,  Combined surgery, LVEF<40% | Fisher exact test | In-hospital | Low |
| Kuitunen 2005[17] | RCT single-center, double-blinded, Finland | 60 cases  N=20 aprotinin  N=20 tranexamic acid  N=20 control | Isolated CABG | Pre-operative anticoagulants or antiplatelet drug | Not specified | In-hospital | Low |
| Landymore 1997[18] | RCT single-center, double-blinded, Canada | 184 cases  N=48 aprotinin  N=56 tranexamic acid  N=44 ε-aminocaproic acid  N=50 control | Isolated CABG  (at least 3 bypass grafts) | Redo operation,  Antiplatelet therapy | Chi-square test | In-hospital | Low |
| Misfeld 1998[19] | RCT single-center, double-blinded, Germany | 42 cases  N=14 aprotinin  N=14 tranexamic acid  N=14 control | Isolated CABG | Not specified | Not specified | In-hospital | Low |
| Mongan 1998[20] | RCT single-center, double-blinded, US | 180 cases  N=75 aprotinin  N=75 tranexamic acid  N=30 control | Isolated CABG | coagulopathy | Chi-square test | In-hospital | Low |
| Lindvall 2008[4] | Retrospective analysis of matched cohort from 2 centres in Sweden (one centre used aprotinin and the other tranexamic acid)  2001 – 2003 | 2,018 cases  Matched paired analysis (matched according to age, sex, and presence of acute coronary syndrome)  N=200 aprotinin  N=200 tranexamic acid | Isolated CABG | Pre-operative clopidogrel in centre using aprotinin | Fisher’s exact test,  Differences between survival curves were analyzed by using the log-rank test | 30-day | Low |
| Karkouti 2010[6] | Retrospective analysis of consecutive series, single centre, Canada  Jan 2000 – May 2008 | 15,534 cases  15,365 analysed  Propensity matched pairs  (5:1 ratio)  Low/high risk:  N=579/ 193 aprotinin  N=577/ 195 tranexamic acid | All types of cardiac surgery with CPB | Patients who did not receive aprotinin or tranexamic acid and those who participated in the BART trial | Multivariable logistic regression  Propensity score adjustment | In-hospital | Subgroup 1: Low  Subgroup 2:  High (predicted risk for major adverse events > 0.3 |
| Sander 2010[7] | Retrospective analysis of consecutive series, single centre, Germany  Jan 2006 – Dec 2006 | 900 cases  Low/ high risk:  N=342/ 215 aprotinin  N=231/ 105 tranexamic acid | All types of cardiac surgery with CPB | Not specified | Multivariable logistic regression | In-hospital | Subgroup 1: Low  Subgroup 2:  High (open heart surgery) |
| Stamou 2009[21] | Retrospective analysis of consecutive series, single centre, US  Jan 2002 - Dec 2006 | 2,101cases  Propensity matched pairs  (5:1 ratio)  N=570 aprotinin  N=114 ε-aminocaproic acid | Isolated CABG (~75-80%), single valve surgery (~15%), combined surgery (~3-8%) | Off-pump CABG, co-administration of both aprotinin and ε-aminocaproic acid | Multivariable logistic regression  Propensity score adjustment  (using proportional hazard Cox model) | In-hospital and 30-day | Low |
| Kristeller 2007[22] | Retrospective analysis of consecutive series, single centre, US  Nov 2003 - Dec 2005 | 742 cases  335 analysed  N=162 Aprotinin  N= 173 ε- aminocaproic acid | Low risk of bleeding:  Isolated CABG ± another surgical procedure (ligation of the left atrial appendage, trans- myocardial laser revascularization, and atrial fibrillation ablation) | High risk of bleeding:  Redo operation,  valve surgery,  aortic surgery,  urgent or emergent operations,  use of clopidogrel or warfarin | Chi-square test | In-hospital | Low |
| Martin 2008[23] | Retrospective analysis of consecutive series, single centre,  Germany  Sep 2005 - Jun 2006 | 1,239 cases  1,188 analysed  Low/ high risk:  N=430/ 166 aprotinin  N=415/ 177 tranexamic acid | Low risk: isolated CABG, single valve surgery  High risk: operations for bleeding, e.g., combined and redo operations, aortic surgery | No antifibrinolytic therapy, multiple drugs, or dose of the antifibrinolytic drug was not sufficient | Chi-square test  Kaplan-Meier analysis and Mantel–Cox log-rank test | 30-day | Subgroup 1: Low  Subgroup 2:  High |
| Shaw 2008[24] | Retrospective analysis of consecutive series, single centre, US  Jan1996 -Dec 2005 | 10,275 cases  N=1,343 aprotinin  N=6,776 ε-aminocaproic acid  N=2,029 control | Isolated CABG (~85%),  CABG+valve surgery (~10%) | Not specified | Multivariable logistic regression  Propensity score adjustment  Kaplan–Meier comparison and Cox proportional-hazards survival analysis | 30-day | Low |

**Table A3: Details of published studies with intermediate risk surgery of in-hospital/ 30-day mortality after cardiac surgery (1990-2012).**

| Later 2009[25] | RCT single-center, double-blinded,  The Netherlands  Jun 2004- Oct 2006 | 333 cases  N=96 aprotinin  N=99 tranexamic acid  N=103 control | Isolated CABG (~30%),  single valve surgery (~30%), combined surgery (~40%) | Redo operation,  antiplatelet therapy, emergency operation | Chi-square test | In-hospital | Intermediate |
| --- | --- | --- | --- | --- | --- | --- | --- |
| DeSantis 2011[8] | Retrospective analysis of consecutive series, single centre, US  Oct 2005 – Oct 2008 | 781 cases  N=325 aprotinin  N=206 tranexamic acid  N=250 ε-aminocaproic acid | CABG ± valve surgery (40-60%), single valve surgery (30-40%), heart transplantation (7-17%) | Congenital surgery ,  LVAD implantation | Multivariable logistic regression  Propensity score adjustment | In-hospital | Intermediate |
| Schneeweiss 2008[26] | Retrospective analysis of prospective database of hospital administration data used for hospital reimbursement (US)  Apr 2003 – Mar 2006 | 162,700 cases  78,199 analysed  Propensity matched pairs (1:1 ratio)  N=4,799 aprotinin  N=4,799 ε-aminocaproic acid | Isolated CABG (~40%),  Complex CABG surgery (~60%) was defined as emergency admission, repeat CABG, or additional cardiac surgery on the day of CABG | Did not receive antifibrinolytic agent, multiple antifibrinolytic agents, received tranexamic acid | Multivariable logistic regression  Propensity matched pairs | In-hospital | Intermediate |
| Mangano 2006[27] | Retrospective analysis of consecutive series,  69 institutions in North and South America, Europe, the Middle East, and Asia | 5,436 cases  4,374 analysed  N=1,295 aprotinin  N=822 tranexamic acid  N=883 ε-aminocaproic acid  N=1,374 control | Isolated CABG (primary surgery; 70%), combined surgery (complex surgery; 30%) | multiple antifibrinolytic agents, inadequate dose of antifibrinolytic agent | Multivariable logistic regression  Statistics for direct comparison aprotinin vs. active (tranexamic acid/ ε-aminocaproic acid) were not calculated | In-hospital | Intermediate |
| Wagener 2008[28] | Retrospective analysis of consecutive series, single centre, US  Jul 2004-Jan 2006 | 428 cases  369 analysed  N=205 aprotinin  N=164 ε-aminocaproic acid | Isolated CABG (~20%), single valve surgery (~30-50%), combined surgery (~20%), redo operation, multiple valve surgery | Off-pump surgery | Not specified | In-hospital | Intermediate |
| Waldow 2009[29] | Retrospective analysis of consecutive series, single centre, Germany  Sep 2006-Mar 2007 | 708 cases  N=369 aprotinin  N=339 tranexamic acid | Isolated CABG (~35%), isolated valve surgery (~30%), CABG+valve surgery (~15%), CABG±valve±aortic surgery (~10%), and others | Redo operation, emergency procedures, pre-operative instability, transplantation | Kaplan-Meyer survival | 30-day | Intermediate |
| Maslow 2008[30] | Retrospective analysis of consecutive series, single centre, US  2000-2007 | 144 cases  123 analysed  N=41 aprotinin  N=82 ε-aminocaproic acid | Isolated CABG (~60-75%),  isolated valve surgery (~10-15%), CABG+valve (~10%), redo operation (~2-10%) | Emergency surgery, pre-operative instability, use of hypothermic cardiac arrest | Fisher‘s exact test | In-hospital | Intermediate |

**Table A4. Details of published studies with high risk surgery of in-hospital/ 30-day mortality after cardiac surgery (1990-2012).**

| Fergusson 2008[1] | RCT, multi-center, double-blind,  19 Canadian centres  Aug 2002-Oct 2007 | 2,331 cases  N=781 aprotinin  N=770 tranexamic acid  N=780 ε-aminocaproic acid | Redo operation (~10%),  CABG+other procedure (~55%), multiple valve surgery, surgery of the ascending aorta or aortic arch | Isolated CABG, isolated valve surgery, congenital heart surgery, heart transplant, LVAD implantation | Multivariable logistic regression | 30-day | High |
| --- | --- | --- | --- | --- | --- | --- | --- |
| Nuttall 2000[31] | RCT, single-center, double-blinded, US | 168 cases  N=40 aprotinin  N=45 tranexamic acid  N=32 tranexamic acid+ autologous blood collection  N= 43 control | Redo operation (CABG, combined surgery) | history of thrombolytic, warfarin, or heparin therapy, Congenital heart disease | Not specified | Not specified | High |
| Wong 2000[32] | RCT, single-center, double-blinded, Canada | 80 cases  N=39 aprotinin  N=38 tranexamic acid | Redo operation, multiple valve surgery, combined procedures, or aortic arch operation | Antifibrinolytic or thrombolytic, or anticoagulant therapy | Not specified | In-hospital | High |
| Jakobsen 2009[5] | Retrospective analysis of consecutive series, single centre, Denmark  Jan 2003 – Dec 2006 | 3,586 cases  3,535 analysed  Propensity matched pairs (1:1 ratio)  N= 534 aprotinin  N= 534 tranexamic acid | Redo operation, multiple cardiac procedures, aortic surgery, high co-morbidity and membership in Jehovah’s Witnesses | Invalid personal identifier, multiple procedures during study period | Multivariable logistic regression  Propensity score adjustment | 30-day | High |
| Sniecinski 2010[33] | Retrospective analysis of consecutive series, single centre, Japan  Jan 2006 - Nov 2008 | 160 cases  N=82 aprotinin  N=78 tranexamic acid | Redo operation,  cardiac surgery requiring deep hypothermic circulatory arrest (aortic surgery (ascending, descending, and/or arch) ± CABG ± valve procedures) | Not specified | Fisher‘s exact test | In-hospital | High |

**References:**

1. Fergusson DA, Hebert PC, Mazer CD, Fremes S, MacAdams C, et al. (2008) A comparison of aprotinin and lysine analogues in high-risk cardiac surgery. N Engl J Med 358: 2319-2331.

2. Henry DA, Carless PA, Moxey AJ, O'Connell D, Stokes BJ, et al. (2011) Anti-fibrinolytic use for minimising perioperative allogeneic blood transfusion. Cochrane Database Syst Rev: CD001886.

3. Karkouti K, Beattie WS, Dattilo KM, McCluskey SA, Ghannam M, et al. (2006) A propensity score case-control comparison of aprotinin and tranexamic acid in high-transfusion-risk cardiac surgery. Transfusion 46: 327-338.

4. Lindvall G, Sartipy U, Ivert T, van der Linden J (2008) Aprotinin is not associated with postoperative renal impairment after primary coronary surgery. Ann Thorac Surg 86: 13-19.

5. Jakobsen CJ, Sondergaard F, Hjortdal VE, Johnsen SP (2009) Use of aprotinin in cardiac surgery: effectiveness and safety in a population-based study. Eur J Cardiothorac Surg 36: 863-868.

6. Karkouti K, Wijeysundera DN, Yau TM, McCluskey SA, Tait G, et al. (2010) The risk-benefit profile of aprotinin versus tranexamic acid in cardiac surgery. Anesth Analg 110: 21-29.

7. Sander M, Spies CD, Martiny V, Rosenthal C, Wernecke KD, et al. (2010) Mortality associated with administration of high-dose tranexamic acid and aprotinin in primary open-heart procedures: a retrospective analysis. Crit Care 14: R148.

8. DeSantis SM, Toole JM, Kratz JM, Uber WE, Wheat MJ, et al. (2011) Early postoperative outcomes and blood product utilization in adult cardiac surgery: the post-aprotinin era. Circulation 124: S62-69.

9. Bernet F, Carrel T, Marbet G, Skarvan K, Stulz P (1999) Reduction of blood loss and transfusion requirements after coronary artery bypass grafting: similar efficacy of tranexamic acid and aprotinin in aspirin-treated patients. J Card Surg 14: 92-97.

10. Blauhut B, Harringer W, Bettelheim P, Doran JE, Spath P, et al. (1994) Comparison of the effects of aprotinin and tranexamic acid on blood loss and related variables after cardiopulmonary bypass. J Thorac Cardiovasc Surg 108: 1083-1091.

11. Casati V, Guzzon D, Oppizzi M, Cossolini M, Torri G, et al. (1999) Hemostatic effects of aprotinin, tranexamic acid and epsilon-aminocaproic acid in primary cardiac surgery. Ann Thorac Surg 68: 2252-2256.

12. Casati V, Guzzon D, Oppizzi M, Bellotti F, Franco A, et al. (2000) Tranexamic acid compared with high-dose aprotinin in primary elective heart operations: effects on perioperative bleeding and allogeneic transfusions. J Thorac Cardiovasc Surg 120: 520-527.

13. Dietrich W, Spannagl M, Boehm J, Hauner K, Braun S, et al. (2008) Tranexamic acid and aprotinin in primary cardiac operations: an analysis of 220 cardiac surgical patients treated with tranexamic acid or aprotinin. Anesth Analg 107: 1469-1478.

14. Diprose P, Herbertson MJ, O'Shaughnessy D, Deakin CD, Gill RS (2005) Reducing allogeneic transfusion in cardiac surgery: a randomized double-blind placebo-controlled trial of antifibrinolytic therapies used in addition to intra-operative cell salvage. Br J Anaesth 94: 271-278.

15. Greilich PE, Jessen ME, Satyanarayana N, Whitten CW, Nuttall GA, et al. (2009) The effect of epsilon-aminocaproic acid and aprotinin on fibrinolysis and blood loss in patients undergoing primary, isolated coronary artery bypass surgery: a randomized, double-blind, placebo-controlled, noninferiority trial. Anesth Analg 109: 15-24.

16. Hekmat K, Zimmermann T, Kampe S, Kasper SM, Weber HJ, et al. (2004) Impact of tranexamic acid vs. aprotinin on blood loss and transfusion requirements after cardiopulmonary bypass: a prospective, randomised, double-blind trial. Curr Med Res Opin 20: 121-126.

17. Kuitunen A, Hiippala S, Vahtera E, Rasi V, Salmenpera M (2005) The effects of aprotinin and tranexamic acid on thrombin generation and fibrinolytic response after cardiac surgery. Acta Anaesthesiol Scand 49: 1272-1279.

18. Landymore RW, Murphy JT, Lummis H, Carter C (1997) The use of low-dose aprotinin, epsilon-aminocaproic acid or tranexamic acid for prevention of mediastinal bleeding in patients receiving aspirin before coronary artery bypass operations. Eur J Cardiothorac Surg 11: 798-800.

19. Misfeld M, Dubbert S, Eleftheriadis S, Siemens HJ, Wagner T, et al. (1998) Fibrinolysis-adjusted perioperative low-dose aprotinin reduces blood loss in bypass operations. Ann Thorac Surg 66: 792-799.

20. Mongan PD, Brown RS, Thwaites BK (1998) Tranexamic acid and aprotinin reduce postoperative bleeding and transfusions during primary coronary revascularization. Anesth Analg 87: 258-265.

21. Stamou SC, Reames MK, Skipper E, Stiegel RM, Nussbaum M, et al. (2009) Aprotinin in cardiac surgery patients: is the risk worth the benefit? Eur J Cardiothorac Surg 36: 869-875.

22. Kristeller JL, Stahl RF, Roslund BP, Roke-Thomas M (2007) Aprotinin use in cardiac surgery patients at low risk for requiring blood transfusion. Pharmacotherapy 27: 988-994.

23. Martin K, Wiesner G, Breuer T, Lange R, Tassani P (2008) The risks of aprotinin and tranexamic acid in cardiac surgery: a one-year follow-up of 1188 consecutive patients. Anesth Analg 107: 1783-1790.

24. Shaw AD, Stafford-Smith M, White WD, Phillips-Bute B, Swaminathan M, et al. (2008) The effect of aprotinin on outcome after coronary-artery bypass grafting. N Engl J Med 358: 784-793.

25. Later AF, Maas JJ, Engbers FH, Versteegh MI, Bruggemans EF, et al. (2009) Tranexamic acid and aprotinin in low- and intermediate-risk cardiac surgery: a non-sponsored, double-blind, randomised, placebo-controlled trial. Eur J Cardiothorac Surg 36: 322-329.

26. Schneeweiss S, Seeger JD, Landon J, Walker AM (2008) Aprotinin during coronary-artery bypass grafting and risk of death. N Engl J Med 358: 771-783.

27. Mangano DT, Tudor IC, Dietzel C (2006) The risk associated with aprotinin in cardiac surgery. N Engl J Med 354: 353-365.

28. Wagener G, Gubitosa G, Wang S, Borregaard N, Kim M, et al. (2008) Increased incidence of acute kidney injury with aprotinin use during cardiac surgery detected with urinary NGAL. Am J Nephrol 28: 576-582.

29. Waldow T, Krutzsch D, Wils M, Plotze K, Matschke K (2009) Low dose aprotinin and low dose tranexamic acid in elective cardiac surgery with cardiopulmonary bypass. Clin Hemorheol Microcirc 42: 269-277.

30. Maslow AD, Chaudrey A, Bert A, Schwartz C, Singh A (2008) Perioperative renal outcome in cardiac surgical patients with preoperative renal dysfunction: aprotinin versus epsilon aminocaproic acid. J Cardiothorac Vasc Anesth 22: 6-15.

31. Nuttall GA, Oliver WC, Ereth MH, Santrach PJ, Bryant SC, et al. (2000) Comparison of blood-conservation strategies in cardiac surgery patients at high risk for bleeding. Anesthesiology 92: 674-682.

32. Wong BI, McLean RF, Fremes SE, Deemar KA, Harrington EM, et al. (2000) Aprotinin and tranexamic acid for high transfusion risk cardiac surgery. Ann Thorac Surg 69: 808-816.

33. Sniecinski RM, Chen EP, Makadia SS, Kikura M, Bolliger D, et al. (2010) Changing from aprotinin to tranexamic acid results in increased use of blood products and recombinant factor VIIa for aortic surgery requiring hypothermic arrest. J Cardiothorac Vasc Anesth 24: 959-963.
