# Supplementary figures and images for: Aprotinin May Increase Mortality in Low and Intermediate Risk but Not in High Risk Cardiac Surgical Patients Compared to Tranexamic Acid and ε-Aminocaproic Acid – A Meta-Analysis of Randomised and Observational Trials of over 30.000 Patients
Source: PLoS One. 2013 Mar 6;8(3):e58009. doi: 10.1371/journal.pone.0058009 (PMC3590293; doi:10.1371/journal.pone.0058009)

**A**

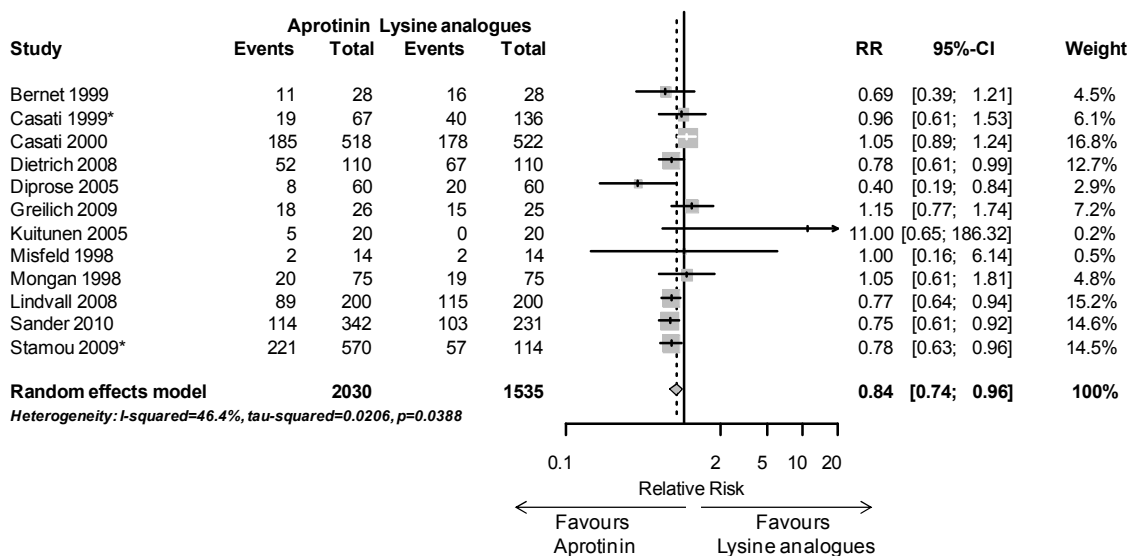

**B**

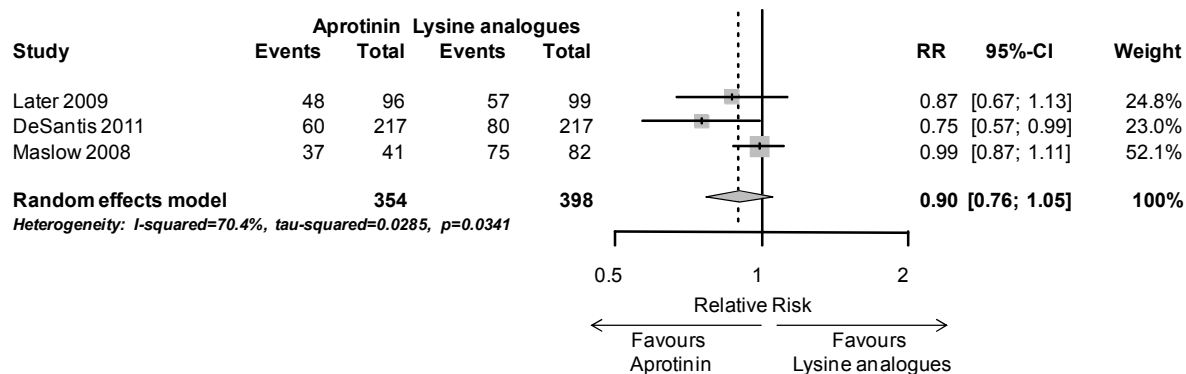

**C**

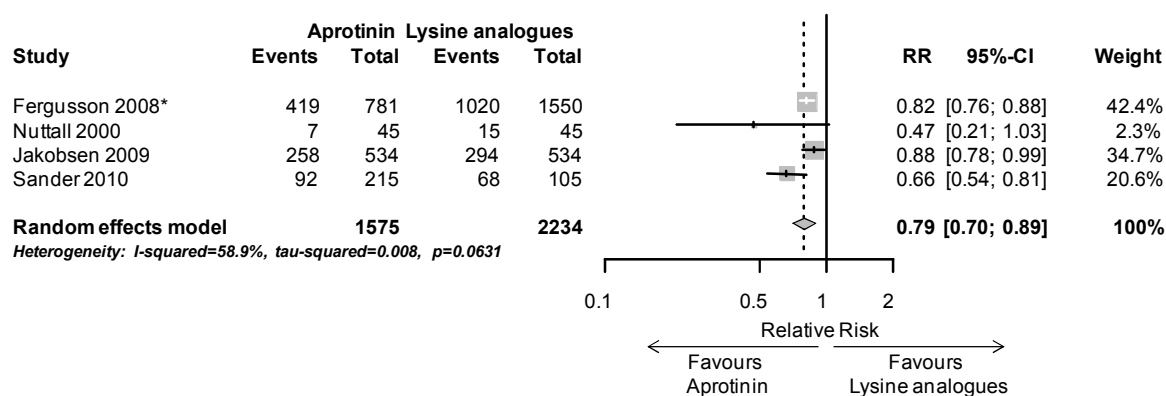

Supplement: Figure S1 — Risk ratio for transfusion of red blood cells within 24 hours after surgery. Forrest plot showing risk ratio (95% CI) of studies comparing aprotinin vs. lysine analogues (tranexamic acid and/or aminocaproic acid, indicated by *) for transfusion of red blood cells within 24 hours after surgery in a subgroup of low (a), intermediate (b) and high risk (c) cardiac surgical patients, respectively. (PDF) [file pone.0058009.s004.pdf]

**A**

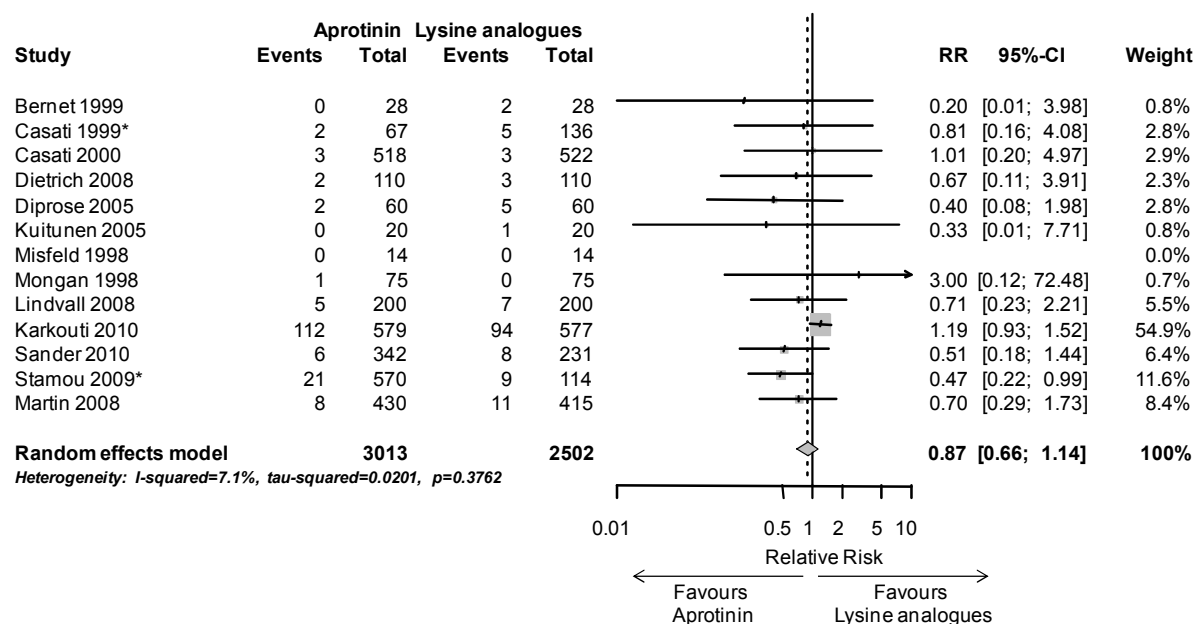

**B**

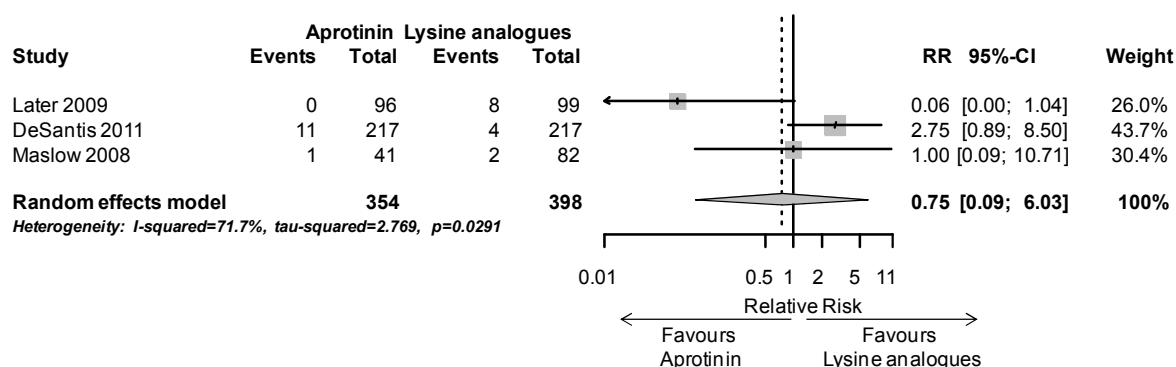

**C**

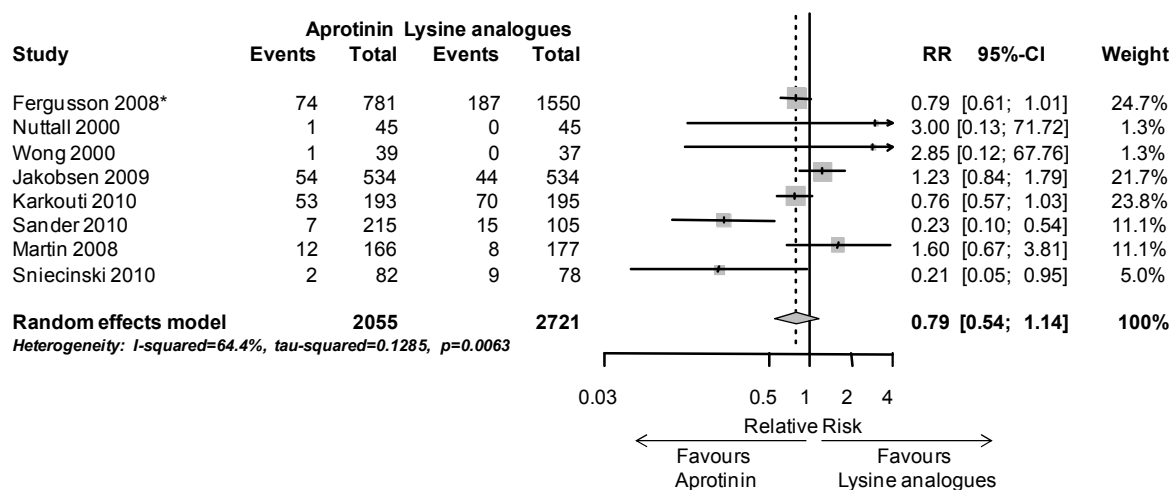

Supplement: Figure S2 — Risk ratio for re-operation or any massive bleeding. Forrest plot showing risk ratio (95% CI) of studies comparing aprotinin vs. lysine analogues (tranexamic acid and/or aminocaproic acid, indicated by *) for re-operation or any massive bleeding in a subgroup of low (a), intermediate (b) and high risk (c) cardiac surgical patients, respectively. (PDF) [file pone.0058009.s005.pdf]

A

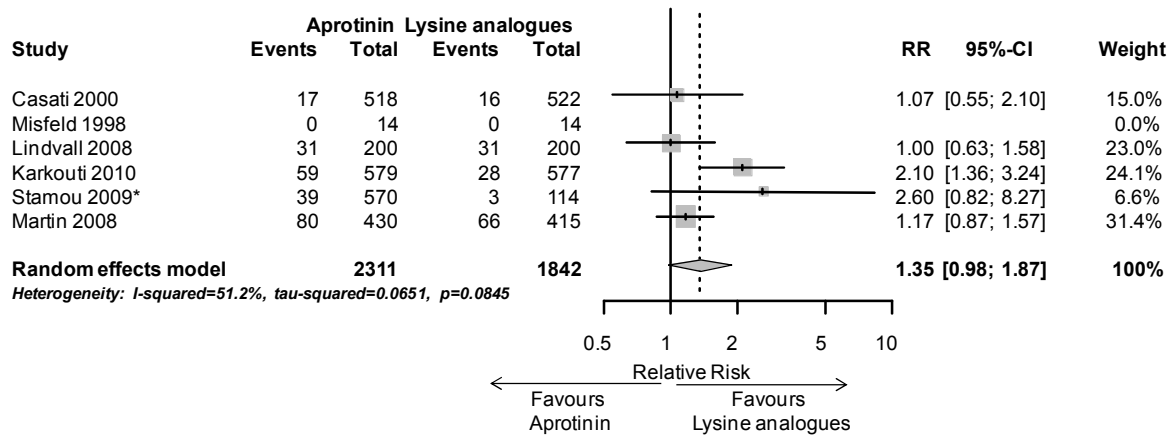

B

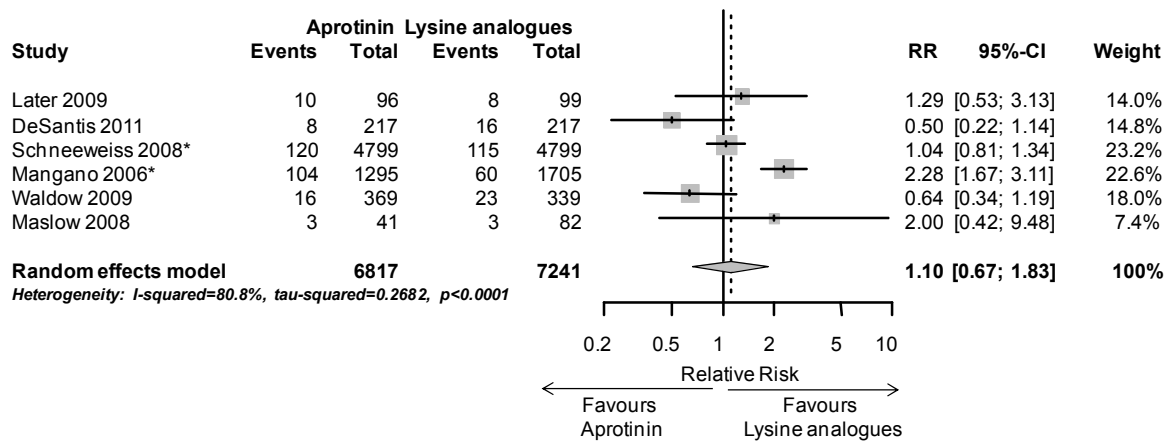

C

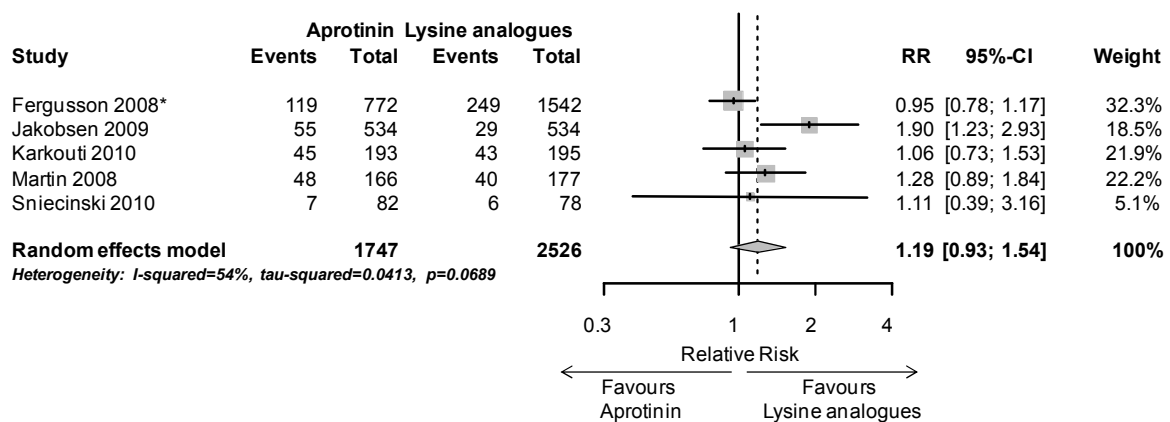

Supplement: Figure S3 — Risk ratio for acute renal dysfunction or acute renal failure. Forrest plot showing risk ratio (95% CI) of studies comparing aprotinin vs. lysine analogues (tranexamic acid and/or aminocaproic acid, indicated by *) for acute renal dysfunction or acute renal failure in a subgroup of low (a), intermediate (b) and high risk (c) cardiac surgical patients, respectively. Please note that definition of acute renal dysfunction and acute renal failure varied moderately between studies. (PDF) [file pone.0058009.s006.pdf]
